# Methotrexate in rheumatoid arthritis is frequently effective, even if re-employed after a previous failure

**DOI:** 10.1186/ar1902

**Published:** 2006-02-24

**Authors:** Theresa Kapral, Tanja Stamm, Klaus P Machold, Karin Montag, Josef S Smolen, Daniel Aletaha

**Affiliations:** 1 Department of Rheumatology, Internal Medicine III, Medical University of Vienna, Vienna, Austria; 2 2nd Department of Medicine, Lainz Hospital, Vienna, Austria; 3 National Institute of Arthritis and Musculoskeletal and Skin Diseases, National Institutes of Health, Bethesda, Maryland, USA

## Abstract

Effectiveness of therapy with individual disease-modifying antirheumatic drugs (DMARDs) in rheumatoid arthritis (RA) is limited, and the number of available DMARDs is finite. Therefore, at some stage during the lengthy course of RA, institution of traditional DMARDs that have previously been applied may have to be reconsidered. In the present study we investigated the effectiveness of re-employed methotrexate in patients with a history of previous methotrexate failure (original course). A total of 1,490 RA patients (80% female, 59% rheumatoid factor positive) were followed from their first presentation, yielding a total of 6,470 patient-years of observation. We identified patients in whom methotrexate was re-employed after at least one intermittent course of a different DMARD. We compared reasons for discontinuation, improvement in acute phase reactants, and cumulative retention rates of methotrexate therapy between the original course of methotrexate and its re-employment. Similar analyses were peformed for other DMARDs. Methotrexate was re-employed in 86 patients. Compared with the original courses, re-employment was associated with a reduced risk for treatment termination because of ineffectiveness (*P *= 0.02, by McNemar test), especially if the maximum methotrexate dose of the original course had been low (<12.5 mg/week; *P *= 0.02, by logistic regression). In a Cox regression model, re-employed MTX was associated with a significantly reduced hazard of treatment termination compared with the original course of methotrexate, adjusting for dose and year of employment (hazard ratio 0.64, 95% confidence interval 0.42–0.97; *P *= 0.04). These findings were not recapitulated in analyses of re-employment of other DMARDs. Re-employment of MTX despite prior inefficacy, but not re-employment of other DMARDs, is an effective therapeutic option, especially in those patients in whom the methotrexate dose of the original course was low.

## Introduction

Rheumatoid arthritis (RA) is a chronic, autoimmune, inflammatory disorder of unknown aetiology that is characterized by symmetric synovitis and the propensity to cause joint destruction, disability and premature death [1-4]. Disease-modifying antirheumatic drugs (DMARDs) slow the natural course of the disease, reduce joint damage and pain, and retard loss of function and disability [5-8].

However, many patients continue to have active disease despite intensive DMARD therapy, or experience adverse events [9,10]. Consequently, within 3–5 years DMARDs must be discontinued in the majority of patients [11-15]. Therefore, changes to therapeutic regimens are frequently required during the chronic course of RA, and many patients receive a large number of sequential DMARD courses [9]. In addition, more rapid switching of DMARDs has become a mainstay in the quest to prevent progression of RA if remission or at least low disease activity cannot be achieved [16,17].

However, the spectrum of traditional DMARDs used in RA is limited. Although the introduction of biological agents has expanded our potential to control RA effectively [18], even these new agents have only limited efficacy in many patients [19-21], and contraindications or reimbursement issues often prevent their use. The spectrum of choices is even more limited, as it has been indicated that combinations of DMARDs are not superior to monotherapy [22,23]. Thus, many patients will reach a point at which their rheumatologists may consider the reinstitution of drugs that have already been employed in the past ('re-employment').

The extent to which re-employment affects the effectiveness and tolerability of DMARDs in RA is not clear. In the present study we investigated the effectiveness and safety of re-employed DMARDs in comparison with the application of the same DMARD in earlier years, identifying patients with a history of prior DMARD failure in a large observational data set. The main focus of our analysis is on methotrexate – the most commonly used DMARD in clinical practice.

## Materials and methods

### Patients

We studied consecutive RA patients at the outpatient clinics of the Vienna General Hospital and Hietzing Hospital, Vienna. Both clinics are specialized referral centres, in which patients are seen at routine outpatient visits usually every three months by rheumatologists or physicians in rheumatology training who have at least one year of clinical experience in the field and are closely supervised by a senior rheumatologist. Since 1997 visits of these patients have been documented prospectively in an observational data set; data from the period between 1989 and 1997 were extracted by chart review. Data quality is ensured by periodical comparison with entries in patient charts and scheduled updates of missing data by data entry personnel. A more detailed description of the data set was published previously [9,11,24].

Briefly, the source data set comprised 1,490 patients who had received at least one course of DMARD therapy, either terminated or ongoing, and had at least one follow-up examination after initiation of DMARD therapy. All patients fulfilled the American College of Rheumatologists 1987 criteria for RA [25]. The data sets of all patients comprised data from their first presentation to our clinics onward, including onset of disease symptoms for determination of disease duration. These patients were aged (mean ± standard deviation) 63 ± 14 years; 80% were female, and 59% were rheumatoid factor positive. Their median (first and third quartile) disease duration at database entry was 5.1 years (1.3 years and 12.3 years). A total of 3,344 DMARD courses were documented, corresponding to a median of two courses per patient (range 1–12). Of the 1,490 patients, 1,227 (82.3%) received their first ever DMARD at our clinics. In these patients, the average delay between disease onset and DMARD initiation was 3.7 years, which is in accordance with the pyramid approach of previous years. For the remaining patients, the DMARD history before first presentation to our clinic was also recorded. In total, we observed 6,470 patient years of DMARD therapy at our clinics. Because methotrexate (although already frequently used in the USA in the 1980s [26]) was not regularly employed in Europe before the late 1980s and early 1990s, the first documented methotrexate therapy was generally also the first application of methotrexate in these patients. Therefore, most patients already had established RA at the time of methotrexate introduction [9].

### Definition, identification and documentation of re-employment

We identified all patients in whom a particular DMARD had been re-employed. 'Re-employment' was defined as the reinstitution of a drug in patients who had a documented course of the same DMARD in their history. To avoid inclusion in the analysis of patients in whom therapy was only temporarily paused and restarted, we also required at least one intermittent course of a different DMARD regimen before re-employment could be declared. Combination therapy including a DMARD previously given as monotherapy was not regarded as re-employment. For each DMARD course, the dosage, duration of therapy, reason for discontinuation (if applicable) and values for erythrocyte sedimentation rate (ESR) and/or C-reactive protein (CRP) at the beginning, during the first year and at the end of therapy were recorded as surrogates of disease activity.

### Outcomes and statistical analysis

We compared three outcomes of DMARD therapy between the original and re-employed courses of methotrexate: reasons for treatment termination; improvement in CRP and ESR at 3, 6, 9 and 12 months after treatment initiation; and treatment retention rates. We also performed these analyses separately for the pooled group of all other DMARDs that had been re-employed. To investigate effects related to a potential dose response to methotrexate, we performed subgroup analyses in patients with higher re-employed doses and those with lower or equal re-employed doses. All statistical analyses were carried out using the Statistical Package for the Social Sciences (SPSS, version 12.0; SPSS Inc., Chicago, IL, USA).

#### Reasons for termination

The two major reasons for termination of DMARD therapy in clinical practice are ineffectiveness (or insufficient effectiveness) and adverse events (insufficient safety) [12,27-29]. Accordingly, we first categorized the reasons for termination of each original DMARD course and each re-employed DMARD course as 'terminated for ineffectiveness' or 'not terminated for ineffectiveness'. Ineffectiveness was defined as persistent or increasing disease activity, in the physician's opinion, despite therapy. We assessed the relationship of this attribute between original and re-employed courses using the McNemar test for paired dichotomous data. To avoid the general exclusion of re-employed therapies that were ongoing at the time of data evaluation (which therefore did not have a reason for treatment discontinuation), we excluded only the subset of those therapies that had been employed for 12 months or less. All currently ongoing therapies that had been employed for more than 12 months were assumed to be effective, and were classified as 'not terminated for ineffectiveness'. In order to conduct a paired analysis, we also discounted all original counterparts of excluded re-employed therapies. In an analoguous manner, we categorized DMARD courses as 'terminated for adverse events' or 'not terminated for adverse events', and performed the same analyses.

There were several other reasons for treatment discontinuation stated in the charts, which were interpreted as follows: lost to follow up and incompliance were categorized as ongoing therapies and were handled as 'not terminated for ineffectiveness' and as 'not terminated for adverse events', respectively, in the two analyses; patients who had been enrolled in clinical trials as the reason for discontinuation were catagorized as 'terminated for ineffectiveness' and 'not terminated for adverse events'; and patients in whom comorbidities were the reason for cessation of therapy were categorized as 'not terminated for ineffectiveness'. If comorbidities were related to therapy, as judged by the evaluator, the therapy was categorized as 'terminated for adverse events'; consequently, comorbiditities that – in the opinion of the physician – were unrelated to therapy (either present before and deteriorating, or newly occurring during treatment) were not regarded as reasons for termination for adverse events.

#### Improvement in acute phase reactants

We obtained values for CRP and ESR as surrogate markers for inflammatory response to therapy [30-33]. We assessed the relative changes in CRP and ESR from baseline at 3, 6, 9 and 12 months after treatment initiation. We then compared these relative changes between original and re-employed courses using the Wilcoxon test for paired nonparametric data.

#### Retention of treatment

Retention of DMARD therapy was assessed using the Kaplan–Meier method, and differences between original and re-employed courses were compared using Log rank statistics. For this analysis, ongoing re-employed courses were censored at the last observed visit. We performed separate analyses of the retention of therapeutic safety in which treatment courses terminated for any other reason than adverse events were additionally censored. In a similar analysis of the retention of therapeutic effectiveness, treatments terminated for any reason other than ineffectiveness were censored. In a multivariate Cox regression model, we assessed whether re-employment status was independently associated with treatment retention, adjusting for methotrexate dose and year of DMARD prescription.

## Results

### Characteristics of patients and treatments

We identified 178 incidences of DMARD re-employment in 163 patients. The median number of DMARDs between original and re-employed course was 1 (range 1–7). There were no patients who had stopped the original courses because of pregnancy or remission. In the 15 patients with two documented incidences of re-employment of the same DMARD (ten MTX courses and five courses of other DMARDs), we only used the first observed event for all analyses to preserve independency of observations. Of the remaining 163 events, 86 included methotrexate and 77 included other DMARDs (Table 1). The group of 'other DMARDs' included mainly sulfasalazine (*n *= 27) and chloroquine (*n *= 25). Rheumatoid factor was present in 59% of all patients in the source data set, and was somewhat more frequent in patients in whom methotrexate or other DMARDs were re-employed (71% and 65%, respectively). By their very nature, the re-employed courses were instituted in late disease. However, the original methotrexate courses were also not applied in early but rather in established disease, mostly after previous DMARD failures. Among the 163 patients, the original courses had been started between 1985 and 2001 and terminated between 1986 and 2002; re-employed courses started between early 1990 and 2002 and were terminated between 1990 and 2004. The mean time interval between the end of original and the start of re-employed courses was 2.1 ± 1.8 years for methotrexate and 2.9 ± 3.1 years for other DMARDs. Concomitant low-dose glucocorticoid treatment was similar among original and re-employed courses (42% and 49%, respectively; *P *= 0.32).

Baseline acute phase reactants were higher for original methotrexate courses than for re-employed courses of methotrexate, but this difference did not reach statistical significance (CRP, *P *= 0.42; ESR, *P *= 0.07). For other DMARDs, baseline CRP and ESR were also similar in both courses (CRP, *P *= 0.75; ESR, *P *= 0.86; Table 1).

### Reasons for termination of methotrexate courses

We analyzed all methotrexate pairs for which the re-employed course had already been terminated or was still ongoing after at least one year of therapy (*n *= 79). The maximum weekly dose was significantly lower with original courses (12.1 ± 4.8 mg/week, median 10.0 mg/week) than in re-employed courses (16.1 ± 6.0 mg/week, median 15.0 mg/week; *P *= 0.0001). The frequency of parenteral methotrexate application was under 10% among both original and re-employed courses.

Among these 79 patients, methotrexate was terminated for ineffectiveness in 51 patients in at least one of the two courses, whereas 19 patients (25.0%) had no evidence of inefficacy in either course. Twenty-three therapies (29.1%) were not ineffective upon re-employment but the original course had been ineffective, while for only nine patients (11.4%) was it *vice versa *(Table 2). This shift toward a lower frequency of ineffective therapies during re-employment was significant (*P *= 0.02, by McNemar test).

**Figure 1:**
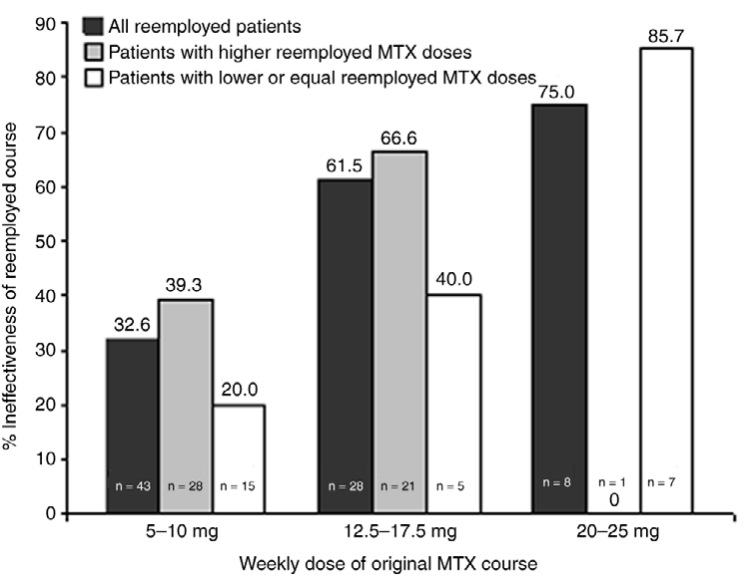
Ineffectiveness of re-employed courses in relation to dose of original course. Bars show the association of treatment termination of re-employed courses for ineffectiveness with the dose of the original methotrexate (MTX) course (total numbers shown in the bars). This relationship is shown for all re-employed courses (black bars; *P *= 0.02, by logistic regression), patients in whom MTX was re-employed at higher doses (grey bars; *P *= 0.43), and patients in whom MTX was re-employed at doses lower than or equal to the original courses (white bars; *P *= 0.01).

Among the 51 patients with inefficacy of the original methotrexate course, the drug was effectively re-employed in 23 (45.1%) patients (Table 2). Although in the original courses in these 51 patients mean doses of methotrexate reached a maximum of 13.3 ± 5.1 mg/week (median 10 mg/week; first and third quartiles 7.5 and 15 mg/week, respectively) before the decision to terminate therapy because of inefficacy was made (Table 2), methotrexate was increased to up to 18.1 ± 5.6 mg/week (median 15 mg/week; first and third quartiles 10 and 20 mg/week) in re-employed courses before termination for ineffectiveness (*P *< 0.001, by Wilcoxon test; Table 2). Figure 1 indicates that discontinuation rates for ineffectiveness of re-employed courses were lower if the methotrexate dose of the original courses had been low: if patients had originally been treated with 10 mg/week or less, then re-employment was ineffective in only about one-third of patients; if they had been treated with a dose greater than 17.5 mg/week in the original course, then the frequency of ineffectiveness upon re-employment was 75%. The effect of the original methotrexate dose on effectiveness of re-employed courses was statistically significant (*P *= 0.02, using Logistic regression). The same effect was observed in subgroups of patients whose re-employed MTX dose was higher than the original dose, and for those whose dose was lower or equal upon re-employment (Figure 1).

We analyzed the same 79 pairs of methotrexate courses with respect to treatment termination for adverse events (Table 3). In 16 (20.3%) of these courses adverse events limited treatment continuation originally but not during re-employment, whereas in 10 (12.7%) it was *vice versa*. There was no statistical evidence that the frequency of adverse events triggering termination of methotrexate was different between original and re-employed courses (*P *= 0.33, by McNemar test), although the methotrexate dose was higher in the 79 re-employed courses (see above). Better tolerance to higher methotrexate doses was probably due to the higher rates of folate substitution in re-employed courses compared with the original courses (35.4% and 24.1%, respectively; *P *= 0.03, by Wilcoxon test). Folate substitution has regularly been instituted since 1995 in original and since 1997 in re-employed courses. When compared with all other subgroups, patients with adverse events in both courses (*n *= 8) had the lowest mean methotrexate doses (Table 3), suggesting that in a significant proportion of patients (about 25%) methotrexate is not tolerated even at very low doses. These patients are therefore possibly more likely to re-encounter adverse effects if methotrexate is re-employed.

In 5.1% of the original methotrexate courses and 1.3% of the re-employed courses reasons of discontinuation were unknown. Among re-employed courses ongoing at more than 12 months from initiation (which were not excluded in the above analyses; *n *= 22), reasons for termination of the original course of methotrexate was classifiable as due to ineffectiveness in 68.2% (*n *= 15) and as due to adverse events in 31.8% (*n *= 7). Among all original courses, treatment termination after 12 months occurred in 36 patients (42%). When the original course was terminated for inefficacy within the first 12 months of treatment (*n *= 22), inefficacy recurred in 59.1% (*n *= 13) of the re-employed course.

### Reasons for termination of other DMARDs

For the group of pairs of all other DMARDs (*n *= 69), there was no significant difference between original and re-employed courses in the frequency of termination due to ineffectiveness (62.3% concordant; *P *= 0.56, by McNemar test) or adverse events (76.8% concordant; *P *= 0.45).

Among these non-methotrexate DMARDs, sulfasalazine was the most frequently used drug (*n *= 23). Four courses were excluded because they were currently ongoing for less than 12 months. The maximum stable dose of sulfasalazine was 2.0 ± 0.6 g/day in original courses and 2.1 ± 0.5 g/day in re-employed courses (*P *= 0.30, by Wilcoxon test). Among patients with ineffective original sulfasalazine therapies (*n *= 16), doses of re-employed SSZ courses were on average similar, specifically 2.1 ± 0.6 g/day before termination for ineffectiveness (*n *= 11) and 2.2 ± 0.5 g/day before termination of re-employed sulfasalazine for other reasons (*n *= 12).

### Improvement in acute phase response

We compared the relative changes in CRP and ESR during the first year of treatment between original and re-employed courses with methotrexate. The improvements in both CRP and ESR throughout the first year were not significantly different between original and re-employed courses (*P *= 0.80 and *P *= 0.43, respectively; Wilcoxon test comparing area under the curves; Table 4). Also, we did not find significant differences in CRP and ESR levels when we compared the subgroups of patients with ineffectiveness in the original and effectiveness in the re-employed course (CRP, *P *= 0.69; ESR, *P *= 0.18) or those experiencing ineffectiveness in both courses (CRP, *P *= 0.58; ESR, *P *= 0.89). In the heterogeneous group of other DMARDs, the re-employed courses were also not associated with significant changes in CRP or ESR (data not shown).

### Retention of treatment

**Figure 2:**
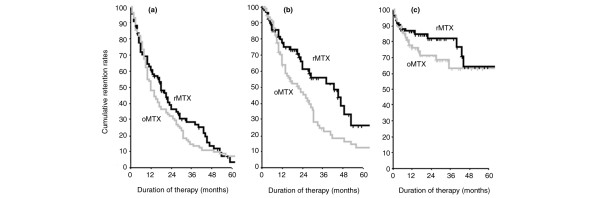
Cumulative retention rates of methotrexate therapies. Kaplan-Meier analysis of retention rates of methotrexate. The different panels show **(a) **the overall drug retention, **(b) **retention of drug effectiveness, and **(c) **retention of drug safety for original therapy (grey lines; oMTX) and re-employed therapy (black lines; rMTX). Overall retention rates were similar between the two groups (panel a: *P *= 0.31, by log-rank test). Likewise, retention because of safety was similar between groups (panel c: *P *= 0.23). Retention because of effectiveness was better for the rMTX group (panel b: *P *< 0.01).

Kaplan-Meier estimates were used to analyze cumulative drug retention rates while accounting for the fact that some re-employed courses were not yet terminated at the time of evaluation ('censoring'). The overall retention rate was numerically slightly longer for re-employed methotrexate (24.3 ± 2.7 months) than for original methotrexate (21.7 ± 2.8 months), but this did not reach statistical significance (*P *= 0.31, by Log-rank test; Figure 2a). However, retention because of therapeutic effectiveness was significantly higher in the re-employed methotrexate group (45.6 ± 5.9 months) than in the original methotrexate group (30.3 ± 4.1 months; *P *= 0.01; Figure 2b), and retention because of safety was slightly higher with re-employed methotrexate courses (87.3 ± 8.6 months) than with original methotrexate courses (70.8 ± 6.2 months), although the latter finding was not statistically significant (*P *= 0.23; Figure 2c).

**Figure 3:**
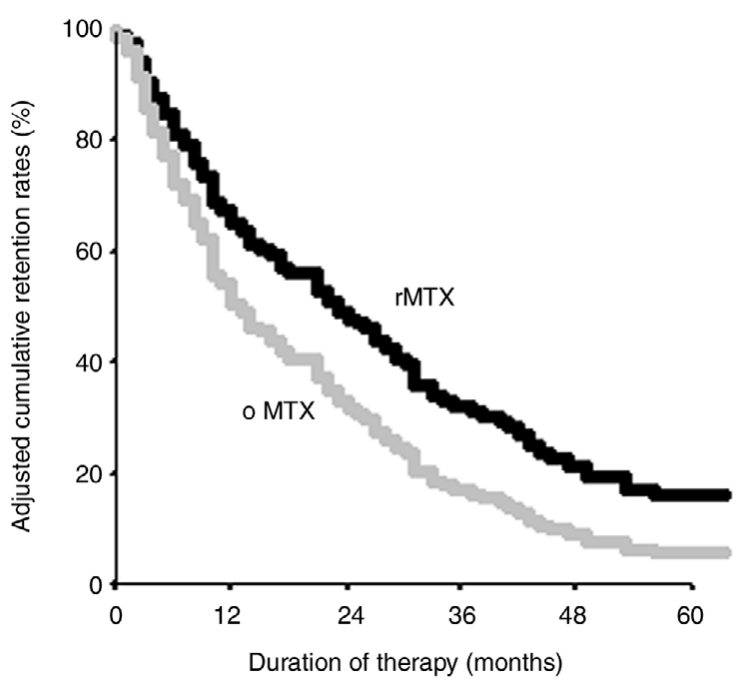
Adjusted cumulative retention rates of MTX therapies. Based on a Cox proportional hazards regression model, cumulative retention rates for original therapies (grey lines; oMTX) and re-employed therapies (black lines; rMTX) are shown adjusted for the use in 1999 or later and methotrexate dose (lines for oMTX and rMTX are plotted at the cohort mean of these covariates). Re-employed courses showed a significant lower Hazard Ratio of treatment discontinuation compared to the original courses (P = 0.04). All courses that were ongoing, lost to follow up, or terminated because of incompliance were censored in this analysis.

Retention rates for therapies might partly be a function of differences in methotrexate dose and the choice of alternatives at the time when a change in therapy is considered [34]. In fact, 54.7% (*n *= 30) of patients were given either biological agents or leflunomide immediately after the re-employed course, whereas this was the case for only 11.3% of original courses (*P *< 0.001). We therefore assessed the independent effect of re-employment on methotrexate retention rates adjusting for methotrexate dose and the year of DMARD prescription (dichotomized at 1999, when the first new DMARDs became available). There was an independent significant benefit of re-employment (using the original therapy as the referent), with a hazard ratio (HR) of 0.64 (95% confidence interval [CI] 0.42–0.97; *P *= 0.04) (Table 5). There was a trend toward better methotrexate retention rates with increasing methotrexate dose (HR per dose increment of methotrexate: 0.97, 95% CI 0.93–1.00; *P *= 0.06). As hypothesized, methotrexate courses prescribed in 1999 or later tended to exhibit a greater risk for termination compared with methotrexate courses prescribed in 1998 or earlier (HR 1.63, 95% CI 0.97–2.73; *P *= 0.06) (Table 5). Figure 3 illustrates the predicted survival functions of original and re-employed courses of methotrexate in the absence of dose differences and independently of the time period in which it had been prescribed.

## Discussion

In the present study we show that rechallenging patients with methotrexate after a prior treatment failure with methotrexate might be a valuable therapeutic option, especially when low methotrexate doses were employed in the earlier course. The rates of termination for ineffectiveness were lower and the adjusted drug retention rates were better for re-employed courses.

Doses of methotrexate were generally higher in re-employed courses. Although it is generally assumed that higher methotrexate doses are more effective than lower ones, there also exists evidence from a recent randomized controlled trial that increasing methotrexate doses in patients with active disease despite methotrexate at 15 mg/week does not improve disease control [35]. Our analysis, however, indicates that rechallenging patients with higher doses of methotrexate in comparison with the original methotrexate course frequently prevented recurrence of treatment ineffectiveness. In particular, effectiveness of methotrexate re-employment was lowest in patients who had been treated with 20 mg/week or more in the original course, but 60% of the patients originally treated with 12.5–17.5 mg/week did not experience inefficacy when treated with equal or lower doses upon re-employment. This was the case in the re-employed methotrexate courses, despite more frequent use of folates, which have been shown to reduce effectiveness of methotrexate [36]. In addition, the patterns of drug retention for effectiveness are different here when compared with those purely related to dose effects, which we previously reported on [11]. In that study, we found essentially no difference in retention of effective methotrexate therapy between the different dose groups; thus, the drug retention pattern of re-employed methotrexate can only partly be explained by higher methotrexate doses in the re-employed courses. Such effects of dose were not seen for sulfasalazine; however, in the context of re-employing sulfasalazine, as well as other non-methotrexate DMARDs, similar doses were used in re-employed compared with the original courses, and therefore a definitive conclusion regarding the potential effectiveness of higher re-employed doses of these DMARDs cannot be drawn. Given our present and previous findings [11] on the role of methotrexate dosing in these patients, it seems that an increase in methotrexate dose should accompany any re-employment of methotrexate, but that a previously unsuccessful course of methotrexate is not necessarily a bad prognostic marker for ineffectiveness of renewed methotrexate treatment at a later stage.

However, our data also indicate that for patients who previously stopped methotrexate because of adverse events, the probability of their recurrence was moderate to high; the lower rate of adverse events seen in two-thirds of patients with previous side effects was probably due to the significantly more frequent use of folates in re-employed courses. We did not analyze the effect of oral versus subcutaneous application of methotrexate on the studied outcomes; more than 90% of our patients received oral MTX. It is still not clear whether parenteral methotrexate provides an advantage regarding effectiveness [35], although it may reduce gastrointestinal side effects during the original course [37].

Given advances in RA therapy accomplished during the past decade by the development and approval of new therapeutic regimens [21], it is also important to evaluate established therapies in situations in which these new drugs might be considered. Most clinical trials require patients to be naïve to the drug to be tested, whereas the typical scenario in clinical practice deals with RA patients who are refractory to a series of DMARDs including methotrexate [9,38]. However, hitherto there are no clinical data available on the potential effect of earlier therapy with methotrexate on the response and future success of renewed methotrexate therapy. This question cannot feasibly be addressed in randomized controlled trials, because the focus of such trials is on direct comparison of two regimens rather than on comparison of one regimen at different times during the course of a patient's disease. We therefore deemed the cohort study design to be the most efficient approach, in which each patient who had received a previously unsuccessful drug served as his/her own control in the analyses when the drug was re-employed.

Given this observational design, the results of the present investigation are prone to bias by indication and secular trends in treatment strategies. Bias by indication includes the notion that clinicians were aware of the methotrexate failure in a patient's history when they decided to re-employ methotrexate, selecting the more refractory patients in whom no other options were deemed valuable. This scenario, however, is precisely that which the study aimed to generalize to. DMARD dose was another difference in the comparison of original and re-employed methotrexate courses. Although the use of increasingly higher doses of methotrexate was in accordance with the secular changes in the state of the art over the past two decades [18], it was important to appreciate the independent effect of re-employment on treatment outcomes. We therefore adjusted for the maximum stable dose reached during the course of therapy using a Cox regression model. Even in this adjusted analysis, there was a significantly better drug retention for re-employed methotrexate compared with original methotrexate. This indicates that re-employment can be a potential option also in patients with sufficient original doses of methotrexate. Thus, most of our findings indicate better outcomes for re-employed courses of methotrexate. This conclusion, however, does not apply to the various other DMARDs, which did not have better effectiveness upon re-employment. Nevertheless, it cannot be excluded that the original course might have been more successful if some DMARDs, especially methotrexate, had been started earlier in the disease. Of interest, approximately 33% of patients on their first methotrexate courses were still on drug at the time of data extraction for the study, which is a further indication of the significant effectiveness of this DMARD in the treatment of RA.

## Conclusion

Reconsidering the use of methotrexate seems to be a rational approach if there was no major toxicity during a previous course of methotrexate. Especially if the methotrexate dose during the initial course was low, re-employment of methotrexate at higher doses may be effective. With regard to new treatment strategies – including monitoring, comedication and even the increasingly employed paradigm to change therapy if a state of low disease activity is not reached within few months [16,17] – re-prescribing methotrexate is an important consideration. This therapeutic option may be valuable in patients in whom other therapies, especially biologicals, cannot be used or have proven insufficiently effective. Thus, the results of this investigation constitute an expansion of therapeutic strategies in the care for RA patients.

## Abbreviations

CI = confidence interval; CRP = C-reactive protein; DMARD = disease modifying antirheumatic drug; ESR = erythrocyte sedimentation rate; HR = hazard ratio; RA = rheumatoid arthritis;

## Competing interests

The authors declare that they have no competing interests.

## Authors' contributions

TK, JS and DA were involved in designing the study, analyzing the data, and drafting the manuscript. TS, KPM and KM were involvement in data acquisition.

## Figures and Tables

**Table 1:** Characteristics of patients and treatments

Parameter	Methotrexate	All other DMARDs
Number	86	77
Age (years [mean ± SD])	60.7 ± 12.9	63.6 ± 11.9
Rheumatoid Factor (% positive)	70.9%	64.9%
Sex (% female)	86.0%	79.2%
Disease duration (years [mean ± SD]; oMTX/rMTX)	9.5 ± 8.3/13.3 ± 8.6	7.5 ± 8.5/12.6 ± 8.6
Prior DMARDs (median [range]; oMTX/rMTX)	1 (0–6)/4 (2–9)	1 (0–8)/3 (2–10)
Baseline dose (mg/week [median (1st-3rd quartile)]; oMTX/rMTX)	10.0 (7.5–15.0)/15.0 (10.0–20.0)	-/-
Base line CRP (mg/l [mean ± SD]; oMTX/rMTX)	33.4 ± 26.2/29.1 ± 31.0	15.2 ± 16.8/18.3 ± 23.0
Base line ESR (mm/hour [mean ± SD]; oMTX/rMTX)	47.0 ± 30.0/35.0 ± 22.0	30 ± 13/32 ± 22

CRP, C-reactive protein; DMARD, disease-modifying antirheumatic drug; ESR, erythrocyte sedimentation rate; oMTX, original methotrexate course; rMTX, re-employed methotrexate course; SD, standard deviation.

**Table 2:** Treatment terminations for ineffectiveness in original and re-employed methotrexate therapies

		Re-employed course		Total
			
		No inefficacy	Inefficacy	
Original course	No inefficacy	19 (9.7 ± 3.1/11.4 ± 4.9)	9 (10.0 ± 4.0/15.8 ± 6.5)	28 (9.8 ± 3.3/12.9 ± 5.8)
	Inefficacy	23 (11.8 ± 4.5/16.8 ± 5.6)	28 (14.5 ± 5.3/18.8 ± 5.2)	51 (13.3 ± 5.1/17.9 ± 5.4)
Total		42 (10.9 ± 4.1/14.4 ± 5.9)	37 (13.5 ± 5.3/18.1 ± 5.6)	79 (12.1 ± 4.8/16.1 ± 6.0)

The numbers in parentheses are the doses in the original methotrexate/re-employed methotrexate courses (mean ± standard deviation) in mg/week). The percentage of patients who were concordant for the presence or absence of inefficacy was 60.3% (*P *= 0.022, by McNemar test).

**Table 3:** Treatment terminations for adverse events in original and re-employed methotrexate therapies

		Re-employed course		Total
			
		No adverse event	Adverse event	
Original Course	No adverse event	45 (13.7 ± 5.2/18.4 ± 5.3)	10 (10.5 ± 3.8/13.5 ± 4.4)	55 (13.1 ± 5.1/17.5 ± 5.5)
	Adverse event	16 (10.2 ± 3.6/14.8 ± 6.3)	8 (8.6 ± 1.3/9.1 ± 2.9)	24 (9.7 ± 3.1/12.9 ± 6.2)
Total		61 (12.8 ± 5.0/17.5 ± 5.7)	18 (9.7 ± 3.2/11.5 ± 4.4)	79 (12.1 ± 4.8/16.1 ± 6.0)

The numbers in parentheses are the doses in the original methotrexate/re-employed methotrexate courses (mean ± standard deviation) in mg/week). The percentage of patients who were concordant for the presence or absence of adverse events was 66.6% (*P *= 0.327, by McNemar test).

**Table 4:** Changes in CRP and ESR with treatment

Time	Change in CRP (*n *= 37)		Change in ESR (n = 20)	
	
	oMTX	rMTX	oMTX	rMTX
3 months	-1.5 (-28.0 and +24.2)	-7.0 (-49.0 and +52.2)	-7.0 (-32.7 and +24.5)	-25.6 (-33.0 and +23.1)
6 months	-13.9 (-59.6 and +16.2)	-11.5 (-49.4 and +22.2)	-20.6 (-50.9 and +19.9)	-12.1 (-38.2 and +52.5)
9 months	-20.9 (-60.1 and +19.0)	-14.8 (-50.4 and +38.4)	-15.3 (-50.6 and +22.2)	-24.2 (-55.3 and +37.6)
12 months	-23.6 (-51.6 and +6.8)	-26.8 (-68.8 and +0.0)	-20.2 (-51.0 and +41.1)	-36.1 (-61.5 and +16.1)
AUC	-14.0 (-41.2 and +16.8)*	-10.3 (-53.0 and +37.9)*	-6.1 (-27.6 and +20.4)**	-17.7 (-45.9 and +25.1)**

Shown are the relative improvements in C-reactive protein (CRP) and erythrocyte sedimentation rate (ESR) at 3, 6, 9, and 12 months after treatment initiation. Values are expressed as median (1st and 3rd quartile) of % change. *P = 0.80, by Wilcoxon test; **P = 0.43, by Wilcoxon test. oMTX, original methotrexate course; rMTX, re-employed methotrexate course.

**Table 5:** Cox regression models on DMARD retention rates

Predictor	Beta (SE)	HR (95% CI)	*P*
Re-employment (reference category: original course)	-0.45 (0.21)	0.64 (0.42–0.97)	0.04
MTX dose (per each 2.5 mg/week dose increment)	-0.03 (0.02)	0.97 (0.93–1.00)	0.06
Employment 1999 or later (reference category: 1998 or earlier)	0.49 (0.26)	1.63 (0.97–2.73)	0.06

In total, 172 were evaluated patients. CI, confidence interval; DMARD, disease-modifying antirheumatic drug; HR, hazard ratio; MTX, methotrexate; SE, standard error.
